# Pneumatosis cystoides intestinalis presenting as pneumoperitoneum in a patient with chronic obstructive pulmonary disease

**DOI:** 10.1259/bjrcr.20230020

**Published:** 2023-06-13

**Authors:** Genna Logue, Mubeen Chaudhry

**Affiliations:** 1 Macclesfield District General Hospital, Macclesfield, United Kingdom

## Abstract

Pneumatosis is always an alarming sign that may result from life-threatening bowel ischaemia and infarction; however, benign intramural gas can also result from a variety of secondary conditions including chronic obstructive pulmonary disease (COPD). Pneumoperitoneum and pneumoretroperitoneum can be seen with both entities. Therefore, thorough discussions with the referring clinicians regarding the patient’s medical history, clinical examination and laboratory results are mandatory. Benign causes can be managed conservatively, however, emergency exploratory laparotomy is often required in suspected life-threatening conditions. Misinterpretation of this finding can lead to incorrect diagnosis and unnecessary surgery.

## Case presentation

A 79-year-old male presents with 1-day history of lower abdominal pain, nausea and vomiting. Biochemical markers of inflammation were raised, and examination findings revealed generalised tenderness with guarding in the left iliac fossa. His past medical history included chronic obstructive pulmonary disease (COPD), large bowel diverticulosis, conservatively managed diverticular perforation and recent acute cholecystitis.

### Investigations

A CT of the abdomen and pelvis enhanced with contrast in the portal venous phase was performed to investigate for acute diverticulitis. Previous CT imaging was available for comparison.

**Figure 1.**
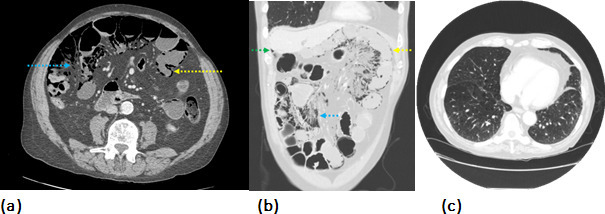
(**a–c**) A 79-year-old male with a history of COPD presents with new onset abdominal pain, nausea and vomiting. An urgent contrast-enhanced CT of the abdomen and pelvis with contrast was performed. (**a**) Axial CT image of the abdomen in the soft tissue window reveals small bowel intramural gas in keeping with pneumatosis intestinalis (yellow arrow) and air dissecting the abdominal fat planes (blue arrow). (**b**) Further demonstration of the aforementioned findings on a coronal reformatted CT image in the pulmonary window, as well as small volume pneumoperitoneum adjacent to the liver edge (green arrow). (**c**) Axial CT image of the chest demonstrated pulmonary emphysema with multiple cystic changes on a background of known moderate obstructive airways disease on spirometry investigation. COPD, chronic obstructive pulmonary disease.

Images demonstrated a small volume pneumoperitoneum within the abdomen and pelvis and associated widespread areas of pneumatosis intestinalis. There were prominent fluid-filled small bowel loops with no signs of bowel obstruction in keeping with an ileus. No portal venous gas was demonstrated. Additional evidence of uncomplicated large bowel diverticulosis and a thick-walled collapsed gallbladder consistent with recent cholecystitis. No free fluid or drainable collection. The visualised lungs demonstrated pulmonary emphysema with multiple small cystic changes (Figure 1).

### Differential diagnosis

Based on the CT findings of diffuse intramural gas and intraperitoneal free gas, a clinical diagnosis of pneumatosis cystoides intestinalis (PCI) with a small volume pneumoperitoneum was made. Differential diagnoses for these radiological appearances include a hollow viscus perforation with associated pneumatosis intestinalis, however, there was no definite site of perforation appreciated on the CT. Correlation with the overall clinical picture and biochemical markers including lactate were advised.

### Treatment

Following initial investigations, the patient was observed by the surgical team. Despite 4 days of intravenous antibiotics, the patient continued to have abdominal pain with mild generalised abdominal tenderness on examination and persistently raised biochemical inflammatory markers.

### Outcome and follow-up

**Figure 2.**
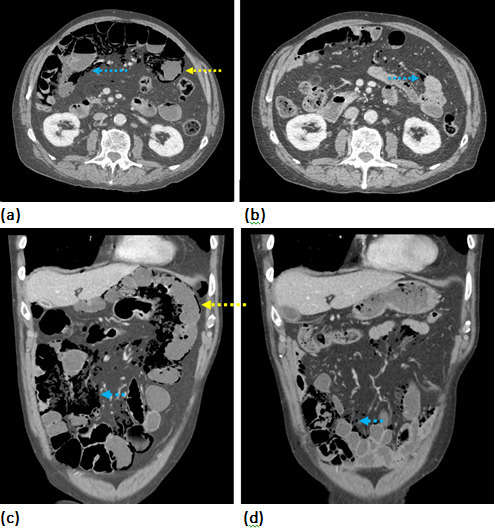
(**a–d**) A 79-year-old male with a history of COPD is investigated with a contrast-enhanced CT of the abdomen and pelvis with contrast enhanced in the portal venous phase which revealed PCI and a small volume pneumoperitoneum. Repeat CT imaging was performed after 5 days of conservative management. (**a**) Axial CT image of the abdomen in the soft tissue window performed as an emergency revealed extensive pneumatosis intestinalis (yellow arrow) and extensive air dissecting the abdominal fat planes (blue arrow). No evidence of bowel obstruction or perforated viscus. (**b**) Axial CT image of the abdomen performed 5 days later revealed interval resolution of the previously described findings and a small volume of residual air within the mesenteric fat (**c**) Redemonstration using reformatted coronal CT images of the abdomen in the soft tissue window performed as an emergency (**d**) and at 5 days. This is in keeping with a benign cause of intramural gas. COPD, chronic obstructive pulmonary disease; PCI, pneumatosis cystoides intestinalis.

A repeat CT of the abdomen and pelvis enhanced with contrast in the portal venous phase revealed less extensive pneumoperitoneum and resolving pneumatosis compared to the CT images performed four days prior. There were no other significant interval changes with no free or localised intraperitoneal fluid collection (Figure 2). After completing a course of antibiotics, the patient was discharged from hospital and recovered from this illness.

In this case, sequel CT examinations excluded a life-threatening cause of intramural gas and supported the diagnosis of this relatively uncommon finding of benign pneumatosis intestinalis. It is often idiopathic and asymptomatic, however when extensive, as with this case, an underlying cause is often present. PCI was felt to be secondary to the patient’s COPD.

## Discussion

The mechanical theory of intramural gas occurs when air migrates from the gastrointestinal lumen into the submucosal or subserosal layer of the bowel wall as a result of increased intraluminal pressure during coughing or intestinal obstruction.^1^ CT findings vary from focal to diffuse gas collections along the gastrointestinal tract forming round cystic collections in the subserosa layer which are often benign features. They can also be found tracking along the submucosal bowel wall creating linear or cresenteric gas patterns; the latter is often, however, not always, indicative of bowel ischaemia.^2,3^

PCI is a rare disease affecting approximately 0.03% of the adult population and is often a self-limited process.^2^ PCI can be divided into a primary and idiopathic aetiology (15%) or a secondary type (85%) which is caused by various predisposing factors including COPD.^3^

The pulmonary theory of PCI involves alveolar rupture and air leakage because of coughing and high airway pressure in COPD. Gas then migrates through vessels within the mediastinum and bowel mesentery before breaching the bowel wall and becoming trapped forming air-filled cyst within the wall.^1^

To our knowledge, we can assume that the latter has occurred in this case. Furthermore, dissection of intramural gas through the serosal layer of the bowel or rupture of a cyst in PCI are hypothetical causes of pneumoperitoneum which result can result in acute abdominal distension and pain.^4^

PCI can occur anywhere within the gastrointestinal tract mainly reported in the large intestine, then small intestine and less frequently in the stomach.^3^ PCI has a vague and broad spectrum of symptoms including diarrhoea, constipation, abdominal distention, nausea, vomiting, weight loss. It can easily be misdiagnosed clinically; however, the radiographic finding of pneumatosis intestinalis should raise the clinician’s concern about the development of life-threatening conditions.^5^

Surgery is only recommended when complications are suspected. The combination of bowel wall soft tissue thickening, free fluid and periintestinal fat stranding and portal venous gas are worrisome features and associated with a life-threatening cause and higher mortality rates.^6^

In benign cases, in the absence of such additional concerning features, the referring clinicians must rely on the patient’s medical history, clinical examination and laboratory results to make life-saving clinical decisions about management. Conservative management includes antibiotics, nasogastric decompression, intestinal rest and oxygen treatment for pulmonary emphysema if appropriate.^2^ Clinical awareness along with close surveillance is vital in the accurate diagnosis and management this condition. Surgical consultation is mandatory if there is any uncertainty or clinical deterioration.^5^

## Learning points

- Intramural bowel gas (pneumatosis intestinalis) is the presence of air within the bowel wall secondary to dysbacteriosis of gastrointestinal flora, poor mucosal integrity, high intraluminal pressure or migration from pulmonary gas.^4,7^
- CT imaging is sensitive for detecting subtle intramural gas, for which the lung window is beneficial. Multiplanar reformatted images enables different patterns of gas collections within the submucosal or subserosal bowel wall to be identified which can help differentiate the underlying pathological process.^7^
- Obstructive airways diseases (asthma and COPD), systemic diseases (scleroderma, systemic lupus erythematosus, AIDS), intestinal inflammation and iatrogenic drugs are some of the causes of benign intramural gas. Yet, this radiological sign also occurs in life-threatening cases of bowel ischaemia and is associated with emergency surgical intervention and a high mortality rate.^4,6^
- Awareness of the causes of intramural gas alongside discussions with the referring clinicians about the patient’s history, examination and laboratory results are critical to avoid misinterpretation, incorrect diagnosis and unnecessary surgery.
